# The power of practice: simulation training improving the quality of neonatal resuscitation skills in Bihar, India

**DOI:** 10.1186/s12887-018-1254-0

**Published:** 2018-09-03

**Authors:** Brennan Vail, Melissa C. Morgan, Hilary Spindler, Amelia Christmas, Susanna R. Cohen, Dilys M. Walker

**Affiliations:** 1 Department of Pediatrics, University of California San Francisco, 550 16th Street, 4th Floor, Box 0110, San Francisco, CA 94158 USA; 2 Maternal, Adolescent, Reproductive, and Child Health Centre, London School of Hygiene and Tropical Medicine, Keppel Street, London, WC1E 7HT UK; 3 Institute for Global Health Sciences, University of California San Francisco, 550 16th Street, San Francisco, CA 94158 USA; 4 PRONTO International, State RMNCH+A Unit, C-16 Krishi Nagar, A.G. Colony, Patna, Bihar 80002 India; 5 College of Nursing, University of Utah, 10 South 2000 East, Salt Lake City, UT 84112 USA; 6 Department of Obstetrics and Gynecology and Reproductive Services, University of California San Francisco, 1001 Potrero Ave, San Francisco, CA 94110 USA; 7 PRONTO International, 1820 E. Thomas Street APT 16, Seattle, WA 98112 USA

**Keywords:** Neonatal resuscitation, Bihar, India, Simulation Training, Barriers to Care

## Abstract

**Background:**

Globally, neonatal mortality accounts for nearly half of under-five mortality, and intrapartum related events are a leading cause. Despite the rise in neonatal resuscitation (NR) training programs in low- and middle-income countries, their impact on the quality of NR skills amongst providers with limited formal medical education, particularly those working in rural primary health centers (PHCs), remains incompletely understood.

**Methods:**

This study evaluates the impact of PRONTO International simulation training on the quality of NR skills in simulated resuscitations and live deliveries in rural PHCs throughout Bihar, India. Further, it explores barriers to performance of key NR skills. PRONTO training was conducted within CARE India’s AMANAT intervention, a maternal and child health quality improvement project. Performance in simulations was evaluated using video-recorded assessment simulations at weeks 4 and 8 of training. Performance in live deliveries was evaluated in real time using a mobile-phone application. Barriers were explored through semi-structured interviews with simulation facilitators.

**Results:**

In total, 1342 nurses participated in PRONTO training and 226 NR assessment simulations were matched by PHC and evaluated. From week 4 to 8 of training, proper neck extension, positive pressure ventilation (PPV) with chest rise, and assessment of heart rate increased by 14%, 19%, and 12% respectively (all *p* ≤ 0.01). No difference was noted in stimulation, suction, proper PPV rate, or time to completion of key steps. In 252 live deliveries, identification of non-vigorous neonates, use of suction, and use of PPV increased by 21%, 25%, and 23% respectively (all *p* < 0.01) between weeks 1–3 and 4–8. Eighteen interviews revealed individual, logistical, and cultural barriers to key NR skills.

**Conclusion:**

PRONTO simulation training had a positive impact on the quality of key skills in simulated and live resuscitations throughout Bihar. Nevertheless, there is need for ongoing improvement that will likely require both further clinical training and addressing barriers that go beyond the scope of such training. In settings where clinical outcome data is unreliable, data triangulation, the process of synthesizing multiple data sources to generate a better-informed evaluation, offers a powerful tool for guiding this process.

## Background

In 2016, 43% of deaths in children under age five globally occurred during the neonatal period [1]. In India, neonatal deaths accounted for 56% under-five deaths [1] and over half of these deaths occurred in only four states: Bihar, Uttar Pradesh, Madhya Pradesh, and Rajasthan [2]. Bihar is a state in eastern India with the highest rural birth rate in the country [3] and the highest multidimensional poverty index in all of South Asia [4]. Nearly one-third of neonatal deaths in Bihar are due to intrapartum related events [5], and yet providers are not adequately trained to perform basic neonatal resuscitation (NR) [6, 7]. Approximately 10% of neonates require tactile stimulation to transition at the time of birth and 3–6% require positive pressure ventilation (PPV) [8]. It is estimated that the effective provision of basic NR could save over 60,000 infants in India alone annually [9].

Although, there are many NR training programs in low- and middle-income countries (LMICs) [10], very few studies have evaluated the impact of such programs on the quality of clinical skills amongst providers with limited formal medical education in rural community settings. One small study evaluating the skills of community health workers in Bangladesh found improvement in initial resuscitation practices (drying, tactile stimulation), neck extension, and mouth-to-mouth ventilation with training, though no statistical analysis was provided [11]. More studies have focused on providers at referral hospitals [12–18]. Results from these studies are variable, with some demonstrating improvements in initial resuscitation [12, 15, 17, 18] and PPV skills [12–17], while others showed no change in initial resuscitation skills [14] or time to initiation of PPV [12, 15]. Several studies assessed skills at one time point and thus could not sufficiently determine the impact of training [19–23]. Others reported only a composite evaluation of skills [24–28], which is less relevant for NR, where outcomes depend on adequate performance of initial steps before proceeding to more complex ones.

This study offers a unique large-scale evaluation of an eight week, in-situ NR training program developed by PRONTO International [29] and implemented in rural primary health centers (PHCs) across Bihar with providers with limited formal medical education. PRONTO training was conducted within a larger maternal and child health quality improvement project called *Apatkaleen Matritva evam Navjat Tatparta* (AMANAT) [30–32]. The specific objectives of this study were 1) to evaluate the impact of PRONTO training on the quality of NR skills in simulated resuscitations; 2) to evaluate the impact of PRONTO training on performance in live deliveries requiring resuscitation of a non-vigorous infant; and 3) to explore obstacles to performance of specific evidence-based practices (EBP) in NR in Bihar.

## Methods

### Study design and setting

This study employed a mixed methods approach to evaluate the impact of PRONTO training on the quality of NR skills. Quantitative methods were used for the first two objectives and qualitative methods were used for the third objective. The portion of PRONTO simulation training evaluated in this manuscript was conducted at PHCs, where the majority of labor and delivery care in Bihar is provided. Each PHC serves a predominately rural population of ~ 190,000 individuals (number based on monitoring and evaluation data from CARE India [30]). PHCs provide largely preventative health care with limited curative services [33]. The vast majority of obstetric and neonatal care at PHCs is provided by nurses with an Auxiliary Nurse Midwife (ANM) or General Nursing and Midwifery (GNM) qualification, which require 2 and 3.5 years of training after completion of secondary school, respectively [34]. PHCs frequently face staffing shortages, often having only one nurse on duty in the labor room at any given time [33]. PHCs are not staffed with specialists, including pediatricians [33], and, in general, doctors are unavailable to assist in the labor room. Caesarian sections and instrumented deliveries are only performed at higher levels of care and thus require referral out of PHCs [33].

### AMANAT and PRONTO interventions

AMANAT is multi-faceted quality improvement project, implemented by CARE India [30] in collaboration with the Government of Bihar, which seeks to improve maternal and child health outcomes in the state using a mentorship model of education [30–32]. AMANAT mentors are nurses with a Bachelor’s degree in nursing recruited from across India. Mentees are ANMs and GNMs employed at PHCs.

PRONTO International training consists of in-situ simulations of a variety of neonatal and obstetric emergencies, which are supplemented by teamwork and communication activities, skills stations, and case-based learning [29]. Within AMANAT, PRONTO was responsible for training mentors to teach mentees emergency obstetric and neonatal care. Doctors were not included in the PRONTO training at PHCs as they were not part of the larger AMANAT program at PHCs and were infrequently involved in labor and delivery care in these facilities. Using a train-the-trainer model, PRONTO provided six days of training for mentors on simulation facilitation, team building, communication skills, and debriefing skills before mentoring began, and a four-day refresher training three months into the mentoring period. Over each 8-month phase, mentor pairs rotated between four PHCs, spending one week per month at each PHC conducting simulations. On average, seven NR simulations were conducted at each PHC over the 8 month training cycle.

In the PRONTO curriculum, normal spontaneous vaginal delivery (NSVD) simulations were introduced in week 2 and NR and postpartum hemorrhage (PPH) simulations were introduced in week 3 of training. Notably, bedside mentoring often began earlier, as mentors attended live deliveries during teaching hours with mentees to provide real-time instruction on any complications that arose. Formal assessment simulations were conducted for NSVD, PPH, and NR at weeks 4 and 8 of training. Pre-training assessments were not conducted, providing mentees time to adjust to simulation prior to being evaluated. NR simulations were conducted with the NeoNatalie™ [35] mannequin in situ in the labor rooms where mentees worked. All simulations were video-recorded to enable video-assisted debriefing as well as for programmatic evaluation.

### Study population

ANMs/GNMs with labor room duties and interest in the mentoring program were selected for participation as mentees in AMANAT and PRONTO training. This analysis evaluates the clinical NR skills of mentees in both real and simulated deliveries in phases 2 and 3 of AMANAT mentoring conducted between September 2015 and July 2016. During this period, approximately 88% of mentees were ANMs and 12% were GNMs.

Interview participants were mentors who served as simulation facilitators. Twenty mentors, one from each phase 4 mentor pair, were selected for interviews in January 2017 based on the following criteria: 1) mentor was currently employed by AMANAT at the time of interview, and 2) mentor had worked in ≥2 phases of AMANAT (equivalent to 16 months in 8 different PHCs). Two interviewees were unable to participate due to illness and personal travel.

### Study procedures

#### Mentee performance in simulated resuscitations

Evaluation of the quality of mentees’ NR skills in simulated resuscitations was based on video-recorded assessment simulations from weeks 4 and 8 of training. At each PHC, mentees were selected by random lottery to participate in the NR assessment simulation for a given week. Assessments were announced but the lottery was conducted immediately prior to simulations. The simulated scenario began with a neonate found apneic while breastfeeding 15 min after birth, progressing to require suctioning, stimulation, and PPV. This simulation was chosen by mentors in place of a simulation beginning with a birth as it involved less set up and was thus easier to facilitate in high volume PHCs. Additionally, it allowed mentees to focus only on NR during the assessment rather than progressing from NSVD management to NR. Assessment videos were transferred to encrypted USB drives and transported to Patna, the capital of Bihar, where they were uploaded to an encrypted server and transferred to University of California San Francisco (UCSF). Videos were then coded by one of the lead investigators with pediatric clinical experience for pre-defined NR quality indicators selected by a team of clinical and simulation experts at UCSF and the University of Utah. The coder was blinded to time of assessment (week 4 vs. 8 of training). After the completion of coding, indicators least likely to be subject to bias due to simulation artifact were selected for inclusion in the analysis. Variable definitions are provided in Table 1.

**Table 1:** Definition of key variables

Binary variables	
Stimulation	Clinically adequate stimulation performed prior to initiation of PPV
Suction	Suction performed prior to initiation of PPV
Neck extension	Neck extended in the proper sniffing position using towel roll or head tilt
PPV with chest rise	PPV with three consecutive breaths with visible chest rise
PPV rate 40–60 breaths/minute	PPV delivered at a rate of 40–60 breaths per minute
Heart rate assessed	Heart rate assessed at any point during the resuscitation
Time-based variables	
Mentee hands on neonate	Time elapsed between the mother calling for help and the nurse mentee placing hands on the neonate to begin the clinical evaluation
Neonate placed on warmer	Time elapsed between the mother calling for help and the neonate being placed on the warmer to begin the resuscitation
Initiation of PPV	Time elapsed between the mother calling for help and the initiation of PPV
PPV with chest rise	Time elapsed between the mother calling for help and the third consecutive breath of PPV with visible chest rise

*PPV* positive pressure ventilation

#### Mentee performance in live resuscitations

Mentors attended births occurring in the PHCs during daytime working hours from Monday through Saturday. Mentors were asked to assess mentees’ skills immediately after observed live deliveries using a smart phone application based on the OpenDataKit platform [36]. The application asked mentors to subjectively evaluate specific NR skills by indicating if the skill ‘went well’ or ‘needed improvement.’ This manuscript only evaluates mentees’ performance during live deliveries in which the neonate was non-vigorous.

#### Barriers to evidence-based NR practices

Mentors were interviewed about the barriers to EBP in NR that they had observed mentees facing in PHCs. Study procedures for the qualitative portion of this manuscript have been described in detail in a separate manuscript [37]. In brief, a semi-structured interview guide was developed and piloted with a former AMANAT mentor. A portion of the interview guide asked mentors about each of the following skills before and after training: warming/drying/stimulating, measuring heart and respiratory rates, achieving chest rise during PPV, and performing the resuscitation with adequate urgency. The interview guide allowed the interviewer the flexibility to ask open-ended questions regarding barriers to these skills and to further explore emerging themes. One-on-one interviews were conducted in English by one of the lead investigators in a private room at PHCs. If the interview was conducted outside of business hours or private space was unavailable, the interview was conducted in a private location near the PHC. All interviewees were fluent in English. Interviews were observed by a local Hindi-speaking member of the PRONTO team in case minor phrase translations were required. Interview duration ranged from 45 to 75 min.

After 18 interviews, the interviewer concluded data saturation had been reached as no new barriers to care were being identified. However, this manuscript only presents barriers specifically linked by mentors to one of the skills evaluated in simulated or live resuscitations in an attempt to provide context for quantitative trends. Thus, this manuscript is not an exhaustive exploration of barriers to care, and other barriers that were not explicitly linked to a specific resuscitation skill are explored in a separate manuscript [37].

### Analysis

All quantitative analyses were conducted using IBM SPSS Statistics 23 [38].

#### Mentee performance in simulated resuscitations

Assessment simulations from weeks 4 and 8 of training were paired by PHC. Simulation videos that were corrupt or could not be paired were discarded. Simulations where the mentor stepped in to assist mentees or where the clinical scenario deviated from the assessment scenario were also discarded. The percentage of simulations in which mentees correctly completed key NR tasks, meeting quality indicators, at weeks 4 and 8 of training was compared using McNemar’s Test for paired proportions. The median time to mentee completion of key NR tasks at weeks 4 and 8 was compared using the Wilcoxon Signed Rank Test due to violation of the normality assumption of parametric methods.

#### Mentee performance in live resuscitations

The percentage of live deliveries in which mentors felt mentees adequately performed key NR skills was graphed by week of training. Additionally, the percentage of deliveries in which NR skills ‘went well’ in weeks 1–3 was compared to weeks 4–8 using the Pearson Chi-Squared Test. Week 3 was chosen as the cut-off because NR simulations were introduced into the PRONTO curriculum at that time. If the expected cell count assumption was violated, a Fisher’s Exact Test was substituted.

#### Barriers to evidence-based NR practices

Audio-recorded interviews were transcribed and analyzed by the interviewer. Qualitative analysis was conducted using the thematic content approach [39, 40], which included 1) data familiarization, 2) identifying codes and then themes, 3) developing a coding scheme and applying it to the data, and 4) refining and organizing codes consistent with the Braun and Clarke approach to thematic analysis [41]. Two interviews (10%) were selected at random for double coding to ensure consistency in identification of key themes.

## Results

### Mentee performance in simulated resuscitations

A total of 1342 mentees at 160 PHCs participated in phases 2 and 3 of AMANAT/ PRONTO training. A randomly selected subset of these mentees was evaluated in 279 NR assessment simulations, which were video-recorded and coded for quality indicators. This analysis includes 226 (81%) assessment videos, or 113 PHC-matched week 4 and 8 video pairs.

From week 4 to 8 of training, there was a 13.5 percentage-point increase in proper neck extension (*p* = 0.01), a 19.0 percentage-point increase in PPV with visible chest rise (*p* < 0.01), and an 11.6 percentage-point increase in assessment of heart rate during resuscitations (*p* < 0.01). There was no statistically significant change between weeks 4 and 8 in adequate stimulation, suction, or delivery of PPV with the proper rate (Table 2). Additionally, there was no statistically significant change in median time to completion of key NR tasks (Table 3).

**Table 2:** Percent of simulations in which mentees correctly performed key NR skills at weeks 4 and 8 of training (*N* = 113 matched pairs)

Key NR skill	N^a^	Week 4	Week 8	Percentage-point change^c^	*P*-value^d^
		*n* (%)^b^			
Stimulation	107	38 (35.5)	26 (24.3)	−11.2	0.08
Suction	111	69 (62.2)	78 (70.3)	8.1	0.25
Neck extension	104	78 (75.0)	92 (88.5)	13.5	0.01
PPV with chest rise	100	66 (66.0)	85 (85.0)	19.0	< 0.01
PPV rate 40–60 breaths/min	106	39 (36.8)	52 (49.1)	12.3	0.08
Heart rate assessed	112	97 (86.6)	110 (98.2)	11.6	< 0.01

*NR* Neonatal resuscitation, *PPV* Positive pressure ventilation

^a^N = total number of PHC-matched week 4 and 8 simulation pairs in which key NR skill could be evaluated

^b^n = number of week 4 and 8 simulations in which key NR skill was completed % = percent of week 4 and 8 simulations in which key NR skill was completed

^c^Percentage-point difference in completion of key NR skill from week 4 to 8 of training

^d^McNemar’s Test of paired proportions

**Table 3:** Time to mentee completion of key NR skills in simulation at weeks 4 and 8 of training (*N* = 113 matched pairs)

Time in seconds to key NR skill	N^a^	Week 4	Week 8	Difference in seconds^c^	*P*-value^d^
		Median (IQR)^b^			
Mentee hands on neonate	98	9 (6–17)	11 (7–22)	2	0.55
Neonate placed on warmer	105	35 (24–56)	38 (26–62)	3	0.95
Initiation of PPV	106	83 (48–111)	84 (66–114)	1	0.90
PPV with chest rise	58	116 (88–178)	137 (92–195)	21	0.76

*NR* Neonatal resuscitation, *PPV* Positive pressure ventilation, *IQR* Inter-quartile range

^a^N = total number of PHC-matched week 4 and 8 simulation pairs in which key NR skill could be evaluated

^b^Median time in seconds to completion of key NR skill (inter-quartile range)

^c^Difference in median number of seconds to completion of key NR skill from week 4 to 8 of training

^d^Wilcoxon Signed-Rank Test

### Mentee performance in live resuscitations

**Fig. 1:**
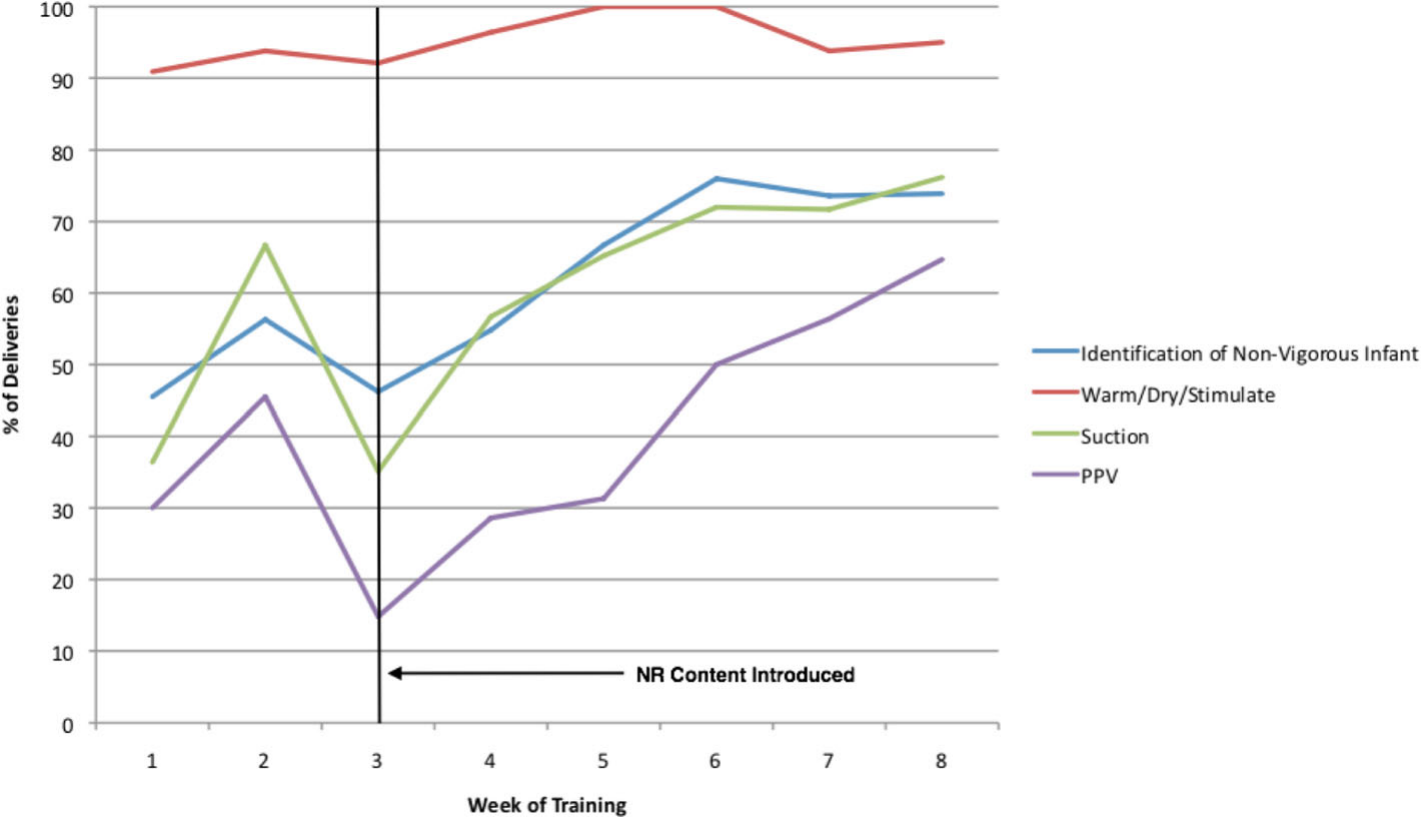
Trend in the Percent of Live Deliveries in which Mentees Successfully Completed Key NR Skills by Week of Training

Mentee performance was evaluated in a total of 3195 live deliveries in phases 2 and 3. Amongst these, 252 (8%) were complicated by birth of a non-vigorous neonate. From early to later weeks of training, the percentage of deliveries in which mentees’ identification of non-vigorous neonates, suctioning, and PPV ‘went well’ increased by 20.7, 25.4, and 22.7 percentage-points respectively (all *p* < 0.01). The percentage of deliveries in which mentors felt mentees performed adequate stimulation was high at baseline (94%) and did not change significantly (Table 4). The week-wise trend in these four variables is illustrated in Fig. 1.

**Table 4:** Percent of live deliveries in which mentees successfully completed key NR Skills in the early versus later weeks of training (*N* = 252)

Key NR skill	Weeks 1–3		Weeks 4–8		Percentage-point change^c^	*P*-value
	N^a^	*n* (%)^b^	N^a^	*n* (%)^b^		
Identification of non-vigorous infant	66	32 (48.5)	156	108 (69.2)	20.7	< 0.01^d^
Warm/dry/stimulate	65	60 (92.3)	144	139 (96.5)	4.2	0.29^e^
Suction	63	27 (42.9)	145	99 (68.3)	25.4	< 0.01^d^
PPV	48	12 (25.0)	109	52 (47.7)	22.7	< 0.01^d^

*NR* Neonatal resuscitation, *PPV* Positive pressure ventilation

^a^N = number of live deliveries in which performance of NR skill was required and recorded

^b^n = number of live deliveries in which NR skill was successfully completed; % = percent of live deliveries in which NR skill was successfully completed

^c^Difference in percent of live deliveries in which NR skill was completed from early to late weeks of training

^d^Pearson Chi-Squared Test

^e^Fisher’s Exact Test

### Barriers to evidence-based NR practices

High level themes and illustrative quotations of barriers to 1) initial resuscitation, 2) measuring heart and respiratory rates, 3) achieving chest rise during PPV, and 4) performing the resuscitation with adequate urgency are summarized in Table 5.

**Table 5:** Barriers to Evidence-Based Practices in Neonatal Resuscitation Before and After Training

Barrier	Before training	After training
Initial resuscitation		
Knowledge	“They were not knowing ok there is a need to stimulate... and they were not knowing ok why they need to dry the baby.”	“So much suctioning is there… with the help of drying or stimulating the baby can be saved, but in spite of that they used to go for suctioning… like if baby didn’t cry means ok get… sucker, get sucker.”
Traditional Practices	“They’ll hold the baby upside down, they will shake the baby here and there, they’ll beat the baby… but… the proper stimulation they were not aware [that] they should rub the baby back or they should flick [the feet].”	
Equipment	“They used to dry the baby but… not with a clean or dry cloth.”	“Baby was [asphyxiated with] thick meconium… suction, all the thing[s] [were] not available and we don’t know where they are.”
Focus on Later Management		“[Mentees] think that if the baby is not crying, they have to take [the baby] immediately to the warmer, so they forget the stimulation part.”
Measurement of heart and respiratory rates		
Knowledge	“Actually before… [mentees] were not knowing ok heart rate and respiration[s] are two different things… then we started teaching them anatomy. Respiration- this is the work of lungs… and heart rate- this is the work of heart.”	
Skill		“[Mentees] don’t have timers to see or… just for name sake they see… or they don’t see it properly… the counting goes here and there. They don’t get it accurately.”
Equipment		“Some sisters [are] having trouble while checking the heart rate because… watch is not available.”
Focus on Later Management	“The goal is the baby should cry. [Mentees] don’t see for the respiration rate or for the heart rate, they just see that the baby cries… keep on stimulating so that the baby cries.”	“Until [mentees] see the baby [cry], they will give bag and mask, bag and mask. In between… check heart rate, respiratory rate, they were not doing.”
Role of MD	“[Mentees] said… ‘what’s heart rate? How do we check that? That’s doctor’s thing, they do that with the stethoscope.’”	
PPV with chest rise		
Knowledge	“They were not knowing about the PPV. If any of the [mentees] knew, she was not knowing the correct rhythm… how much time you need to do, how you need to. She only knew ok we need to do.”	
Skill	“[Mentees] just pump [the Ambu bag]… according to the baby[‘s] size they don’t use the [correct] mask. Whatever mask they get, they will connect that and they will pump it.”	“[Mask] seal is not good for most of the time… and the rhythm also. Some of the mentees, they forget the [ventilation] rhythm also.”
Traditional Practices	“Before… in some facilities [mentees] were giving mouth to mouth ventilation… that time they didn’t know how to use bag and mask ventilation.”	“PPV they are doing but they have more belief in oxygen. If we will put the oxygen… baby will be crying they believe only.”
Equipment		“In some PHC we don’t have zero [size] mask… we have only one number mask, so it is not as effective, because in preterm baby we can’t use the big one.”
Role of MD	“Before training [mentees] were not doing [PPV]… they didn’t know how to use bag and mask ventilation. They only know… we can’t use, doctor has to do.”	
Urgency		
Knowledge	“Actually they are not aware what is the effect [of delay]. Until we… know what is the effect, we will not take precaution.”	“[Mentees] can’t… understand when [the neonates] need resuscitation or not. Sometimes they identify very well but… sometime[s] they waiting for… crying… It’s not proper timing.”
Skill	“To cut the cord, to take the baby to the NBCC, and to start [the] resuscitation, it will take more than 5 min they were telling.”	“It will take time, especially drying the baby, wiping it, stimulating it, clamping… the cords.”
Traditional Practices	“Because their old practice is like... they… will wait, they’ll tell, ‘Baby will cry now, sister this is normal baby will cry now.’”	“They are thinking it might be crying… they are waiting for some time. But when we are there we are telling them not crying so go fast!”
Equipment		“Golden minute… [mentees] don’t have articles for clamping or… they search for suctioning, for mucus extractor… availability is not there in the PHC, so they go outside to get.”
Facility Layout		“NBCC is in another room… this is labor room, so next to labor room is NBCC, so that takes [mentees] more than a minute to take the baby from labor room to NBCC.”
Maternal Management	“For one to two to three minutes [mentees] will wait… because [until] the placenta is removed, they will concentrate on that. Ok, the placenta is removed, after that they see, ok, baby is not crying. Then they will start with the Ambu.”	
Human Resources		“Sometimes only one staff is there for delivery… she will be taking care of the mother and then baby is not crying...”

*PPV* Positive pressure ventilation, *PHC* Primary health center, *NBCC* Newborn care corner

#### Initial resuscitation

Prior to training, mentors explained mentees did not understand the clinical significance of the initial steps of resuscitation (warming, drying, stimulating, and suction) and did not know how to properly perform these steps. Rather, they performed traditional practices including holding the neonate upside down, over stimulating, and massaging the chest. Additionally, equipment issues, including the availability of clean, dry cloths precluded effective initial resuscitations.

After training, mentors felt that mentees knew how to perform warm/dry/stim in an evidence-based manner. However, mentors reported that mentees often forgot to perform these initial resuscitation steps in a perceived rush to begin ventilation. On the other hand, mentors felt mentees still did not understand the clinical indications for suctioning and were too quick to jump to this step. Supply issues remained a barrier to initial resuscitation after training. Mentors explained that equipment, including mucus extractors, was often unavailable or disorganized and thus inaccessible when urgently needed.

#### Measurement of heart and respiratory rates

Mentors explained that prior to training, mentees did not know how to measure vital signs, were inaccurate in their counting, or were unaware of normal parameters and their clinical significance for neonates. This was likely connected to the belief, prior to training, that the management of non-vigorous neonates was the responsibility of doctors. Mentors also explained that mentees’ goal in resuscitations was simply to make the baby cry, so vital signs were frequently overlooked.

This goal remained true after training. Mentors reported that mentees frequently forgot to check vital signs because they were too focused on simply making the neonate cry. Nevertheless, mentors felt that mentees understood the significance of vital signs after training. However, they still could not measure them accurately, often because they did not have or could not read a clock.

#### PPV with chest rise

Mentors explained that knowledge of all aspects of PPV, including clinical significance, mask selection, rate of delivery, and assessment of effectiveness was lacking before training. If ventilation was provided, it was often given mouth-to-mouth or by using a self-inflating bag on the mother’s abdomen without knowledge of proper technique. Similar to the measurement of vital signs, mentors explained that some mentees believed that doctors were responsible for managing non-vigorous neonates prior to training, which meant they did not initiate ventilation themselves.

After training, mentors felt mentees had accepted the responsibility of providing PPV, but that they continued to have difficulty with mask seal, rhythm, and assessment of PPV effectiveness. Approximately two-thirds of mentors reported observing continued difficulty with neck extension after training, while one-third of mentors felt mentees had mastered this skill. Additionally, mentors reported mentees did not know when to stop PPV for reassessment because mentees did not have or could not read a clock. The availability of ventilation bags and different mask sizes, particularly preterm masks, was identified as a barrier after training-- likely persistent from before training but more frequently identified after PPV became an accepted duty of mentees. Finally, one mentor felt the traditional belief that oxygen was important in addressing respiratory distress was a barrier to performing PPV with self-inflating bags with no oxygen source after training.

#### Urgency

Mentors explained that mentees did not understand the concept of the golden minute or the significance of achieving effective ventilation within that timeframe prior to training. Additionally, they did not know how to accurately identify non-vigorous neonates requiring resuscitation. Further, mentors explained the traditional practice in Bihar was to patiently wait for neonates to cry, which commonly delayed resuscitations. Other delays were created by slow cord clamping and performance of the initial NR steps. Finally, mentors described mentees’ focus on maternal management as a barrier to timely NR prior to training.

After training, mentors explained mentees were better at identifying non-vigorous neonates and knew about the golden minute; however, some mentors expressed concern some mentees still did not truly understand its clinical significance. Additionally, mentors explained mentees could not read a clock to facilitate timely resuscitations. Regarding skills, mentors explained mentees’ inefficiencies in initial resuscitation and cord cutting continued to delay resuscitations after training. One mentor felt that mentees spent too much time trying to seal the mask. Overall, mentors felt more practice performing NR with proper timing was necessary. Other frequently mentioned barriers to urgency that were likely persistent from before training were the traditional practice of patiently waiting for the infant to cry, long distances between labor rooms and the newborn care corners (NBCCs), insufficient staffing, and issues with supply availability, functionality, and organization.

## Discussion

PRONTO International’s NR simulation training, implemented within the AMANAT quality improvement initiative, had a positive impact on key NR skills amongst ANM/GNM mentees working in rural PHCs across Bihar. Nevertheless, there is room for continued improvement in nearly all NR skills, likely due to the need for additional training as well as significant barriers that go beyond the scope of clinical skills training. For each of the key skills evaluated in this manuscript-- initial resuscitation, assessment of vital signs, performance of PPV, and urgency in resuscitations-- we present a triangulated discussion of simulation data, live delivery data, and barriers to care identified by mentors in qualitative interviews to facilitate a more nuanced understanding of the positive impacts of PRONTO training and areas for improvement.

Mentees’ performance of the initial NR steps, including warming, drying, stimulating, and suctioning, was variable. This is consistent with previously published studies [12, 14]. In interviews, mentors suggested that knowledge of EBPs increased with training. However, there was no significant change in the percentage of simulated NR scenarios in which mentees provided clinically adequate stimulation prior to PPV from week 4 to 8 of training. In observed live deliveries, there was similarly no significant change in stimulation between the early and later weeks of training; although, the rate of stimulation was high at baseline. This knowledge-skill gap may be explained by mentors’ observation that mentees frequently forgot initial NR steps in a perceived rush to start PPV. Moreover, the fact that the simulated scenario did not begin with a birth may have also contributed to mentees’ relative failure to perform initial steps in simulation compared to live deliveries. Regarding suctioning, there was significant improvement in live deliveries, but not in simulated resuscitations. Despite this improvement in live deliveries, about a quarter of live-born neonates deemed to require suctioning did not receive it during week 8 of training, perhaps due to the supply issues highlighted by mentors.

Assessment of vital signs, including heart rate and respiratory rate, was evaluated only in simulated resuscitations. A significant improvement was observed from week 4 to 8 of training. Mentors explained that vital signs were often not assessed before training due to inadequate knowledge and a prevalent belief that NR was the doctor’s responsibility. This suggests the observed change in simulation data, which did not include a true pre-training measurement, may underestimate the impact of training on this skill. Notably, while simulation data captured whether or not mentees checked heart rate, it did not assess the accuracy of heart rate measurements. Mentors explained in interviews that mentees have difficulty reading a clock, suggesting this may be an area for future improvement. This will likely require innovative solutions to help providers identify normal versus abnormal vital signs without the need to count precise rates.

Proper delivery of PPV is the chief focus of many NR trainings. A significant improvement in PPV skills was observed in both simulated and live resuscitations following PRONTO training. Previous studies have similarly reported improvement in PPV skills post-training [12–17]. During week 8, mentees achieved chest rise in 85 and 65% of simulated and live resuscitations, respectively. Other studies report comparable [12, 15] or lower rates of effective PPV [14, 16]. In interviews, mentors explained mentees continued to struggle with mask seal, rhythm, and real time assessment of PPV effectiveness. These observations are supported by the simulation data, which demonstrated no change in the use of the proper rate of PPV following training. Although interviewees disagreed about mentees’ ability to perform proper neck extension, a significant improvement in this skill was observed in simulations from week 4 to 8. Mentors felt the persistent PPV knowledge-skill gap was due to insufficient practice as well as lack of availability of functional supplies in PHCs. The need for more practice with longer trainings is not an unfamiliar challenge amongst NR programs in LMICs [42] and the PRONTO training is unique in that it was conducted over 8 months. Nonetheless, given the departure PPV represents from traditional practices in Bihar, interviewees felt even this duration of training was insufficient.

Urgency is another key area for improvement. No significant change was observed in the time to completion of key NR tasks in simulations. In fact, the median time to effective chest rise trended upward non-significantly from week 4 to 8 of training. Other studies have similarly reported both increased and unchanged durations of time to PPV initiation [12, 15]. Nonetheless, mentors described a perceived rush to start ventilation after training that negatively impacted initial resuscitation measures. The discrepancy between the perceived urgency and true time to completion of key tasks may be related to barriers such as inability to read a clock, distance between labor rooms and NBCCs, and both supply and human resources shortages. Other barriers to urgency identified by interviewees included poor understanding of the true significance of the golden minute and continued performance of traditional clinical practices such as waiting indefinitely for the infant to cry. Timely identification of non-vigorous neonates in live deliveries improved significantly; however, mentees still failed to identify nearly a quarter of live-born neonates deemed non-vigorous by mentors at the end of training.

These results have informed the next iteration of the PRONTO curriculum, which will include greater emphasis on quick identification of non-vigorous neonates, beginning resuscitations with appropriate initial resuscitation measures, recognition of vital sign abnormalities without counting specific rates, and timely initiation of effective PPV. Nevertheless, this study has several limitations. Foremost, due to the unreliable birth registry system in Bihar, there are no reliable clinical outcome data on which to base the impact of this training program. For this reason, we used simulation data as a proxy.

The simulation data lack a true pre-training measurement, which may cause an underestimation of the true impact of training. Nonetheless, this was a conscious choice to allow mentees to adapt to simulation procedures prior to evaluation given their lack of familiarity with this method of learning [42]. The assessment simulation was also not changed between week 4 and 8. However, this is unlikely to have led to an overestimation of the impact of training given the aim of this study was to assess the quality of basic NR skills, which should follow an algorithm that is relatively independent of the clinical scenario in uncomplicated resuscitations. Additionally, simulation data represent the performance of only a subset of mentees who participated in the NR assessment simulations at week 4 and 8 of training. However, as the selection process was random, the impact of selection bias is likely minimal. Finally, this data is based on a single video assessor, which could have introduced interpretation bias. However, the potential for this bias was minimized by blinding the assessor to week of training and by choosing an assessor who was independent from training implementation.

The live birth data represent a convenience sample and could be biased, as data were collected by mentors who were not blind to week of training and who had somewhat limited clinical training themselves, as most were early in their nursing career. Further, live delivery data provide only a binary and subjective assessment of whether key NR steps went well or not. Nevertheless, these data provide the only assessment of performance in live deliveries, as medical record keeping is inconsistent. The investigators felt that a more rigorous assessment of resuscitations in real time would impact clinical care or preclude data collection given the high delivery volume at PHCs.

Qualitative interview data could be influenced by desire of mentors to please the interviewer as well as by any preconceptions mentors may have had about intrapartum or postnatal care in Bihar. We attempted to mitigate these potential biases by clearly stating during the consent process that interviews were not a performance evaluation and by selecting interviewees with at least 16 months of mentoring experience in PHCs. Finally, not all qualitative interview data regarding barriers to care is included in this manuscript. Rather, logistical, cultural, and structural barriers to immediate neonatal care and NR are more fully explored in a separate manuscript [37] and this manuscript only presents barriers explicitly linked by mentors to specific NR skills assessed in simulated and live resuscitations.

## Conclusion

PRONTO simulation training conducted within the AMANAT intervention had a positive impact on knowledge and the use of evidence-based NR practices amongst numerous ANMs/GNMs working in rural PHCs throughout Bihar. Nevertheless there is a need for ongoing improvement, which will require addressing many barriers to care that extend beyond the scope of clinical skills training. Data triangulation, incorporating both quantitative and qualitative methodologies, offers a powerful tool for guiding this process in settings such as Bihar where clinical outcome data are unreliable, yet the need for improvement in neonatal care is great.

## Abbreviations

ANM: Auxiliary nurse midwife
EBP: Evidence-based practices
GNM: General nursing and midwifery
IQR: Inter-quartile range
LMIC: Low- and middle- income countries
NBCC: Newborn care corner
NR: Neonatal resuscitation
NSVD: Normal spontaneous vaginal delivery
PHC: Primary health center
PPH: Postpartum hemorrhage
PPV: Positive pressure ventilation
UCSF: University of California San Francisco
